# Isopathic and Other Pathological Prescribing

**Published:** 1891-05

**Authors:** 


### ISOPATHIC AND OTHER PATHOLOGICAL PRESCRIBING.

It seems to be an inherent tendency in human nature to be always seeking to find easy, short methods to accomplish difficult tasks; and this tendency is surely to be commended if success be not sacrificed in the endeavor. As far as the tendency is exerted in medicine it is very often followed by failure to secure the best curative results. Years ago, Hahnemann gave to the medical world his *Organon*, and showed what could be done for the relieef of the sick by a strict application of the *Law* of the *Similars*. This is a law of Nature, not of man; a law by which man can attain to almost mathematical certainty in his prescribing and curing. But it is very difficult to apply this law in its strictest sense; hence we weak, fallacious creatures are always seeking to make it easier, and not always in a line to secure success in curing at the same time. The only true way to secure success in healing, and to lighten our task in prescribing, is to perfect our materia medica, to learn to be skillful in examining our patients, and to know the cardinal principles of homœopathic practice. One must know his materia medica, must know how to examine a patient, must know which symptoms are to be used in selecting the remedy, and lastly, but by no means least, must know how to give the selected remedy. These few thoughts upon the subject of homœopathic prescribing are so trite, and have been mentioned so frequently, that one is almost ashamed to waste space upon them again; yet they do not seem to be understood or appreciated.

The true method of homœopathic prescribing is to select for each patient a drug whose characteristic symptoms are most similar to those of the patient. This is called prescribing by Symptomatology. It is difficult to do this correctly, for many reasons; in the first place, we do not know, as well as we should, the characteristic symptoms of our drugs; in the second, it is difficult to always obtain the true characteristic symptoms of each patient. At any rate, it is a laborious task to examine each patient, to find all his symptoms, and then to select from the materia medica that remedy which is most similar. Those physicians who have followed this laborious path declare it leads to successes that are simply marvelous; those who have not followed this laborious path declare it leads one into foolish errors and worse than all, leads him away from the fashionable theories of the day! A terrible mistake in their opinion!

A recent writer in the *New York Medical Times* (for March, 1891, p. 362), is very much disturbed over the old-fogy errors of Homœopathy, and pleads most earnestly for us to abandon our evil ways. That old bugbear, psora, troubles him very much; he insists on calling it the "itch," and declares Hahnemann taught that all chronic ailments are products of this "itch." If that name does not suit you then choose another, my friend. The chronic miasm, or dyscrasia, or what not, will be just as active (unfortunately for us) under one name as another. Our friend asks: "Can it be possible, that our school of medicine will longer persist in harboring such untruth, such nonsense, in the bright light, the purer light, the microscopical light, which characterizes the close of this nineteenth century?" * * * "We need to be disinfected. It is high time to clean house, and get rid of that which is offensive to others and detrimental to ourselves."

It is just because the pathological light of this nineteenth century is *microscopic* in its truth that we decline to give up our facts for its fancies. We do not ask whether it be offensive to others or not, we merely seek to know if it be true or false. If our friend has kept his eyes open and has observed the pathological changes which occur every day among the patients treated by allopathic physicians, he has, doubtless, noticed many cases proving the active presence of this psora, or dyscrasia. Has he never seen a young, blooming maiden, healthy and strong up to the day of her marriage; never needing a gynæcologist until, maybe after her first child is born, then some slight pelvic disturbance calls for "*an examination.*" This examination shows some slight ailment which must be treated locally; it is done, and that woman is thereafter never out of the hands of the gynæcologist. The first trifling ailment is "treated" (that is, *suppressed*), the woman is well for awhile; soon she complains again, this time of a worse pain or weakness, etc., she is again examined, treated, is "well" again; next year she complains again and goes through the same routine, which ends with an operation for fibroids, for a sarcoma, for an ovarian cyst, etc. If our friend has seen such a course of pathological conditions, then he has observed that which Hahnemann called psora, or suppressed disease action. Did he ever observe a simple nasal catarrh, or a simple laryngitis treated by a laryngologist finally end in death by phthisis? Did he ever see a simple eruption treated (that is, driven in) by a dermatologist, end in some nervous disorder like epilepsy or insanity? There is, to-day, a young woman in an asylum near this city, and she has been there these five years, who, her allopathic physicians say, was made insane by over-dosing with narcotics used to relieve neuralgic toothache.

If our friend has ever observed any of these, or numerous other evidences of suppressed disease action, then he has seen that which Hahnemann called psora. An unfortunate name, perhaps, but one that expresses a dire fact for suffering humanity; a fact which, unfortunately, cannot be abolished by sneers or frowns.

After the bugbear, psora, the error of prescribing without a diagnosis disturbs our friend. He says: "Brothers, in all sincerity and brotherly love, I say it is hazardous to prescribe without a *reasonable* diagnosis; and, also, hazardous to prescribe with an erroneous diagnosis." One might well ask our friend how he is to prevent a "reasonable" diagnosis from becoming an "erroneous" one? Furthermore, we are told that "Similia is broad, but it has its bounds, and there are places in which it is inoperative; but the most perfect similia takes in etiology and pathology, as well as symptomatology, and thus escapes the blunder of many a misapplication. As long as there is one pathological condition in which Homœopathy is inoperative, it is hazardous to prescribe without a diagnosis."

All this is an old, old story to Hahnemannians; they all know full well that prescribing upon diagnosis is fallacious, misleading; that it is simply replacing law by theory, fact by fancy; that it has been tried and found wanting. It is equally fallacious whether the drug used be given in a CM potency or in the crude state. This is the weak spot, the error of so-called Isopathy; it is essentially prescribing upon a diagnosis which is too often an "erroneous" one and hazardous.

The isopathists claim that the specific, morbific cause of diseases will, when potentized, cure those diseases; that such diseases as syphilis, gonorrhœa, diphtheria, scarlet fever, typhus, erysipelas, itch, septicæmia, scirrhous, cancer, phthisis, and glandular diseases (what are "glandular" diseases?) can be cured by a potentized form of their morbific cause. If this be true then Homœopathy is false, for it teaches that, under the Law of the Similars, there is only one way of treating all cases of disease, which consists in proving drugs upon the healthy and prescribing them for such symptoms as each patient may present. Potentization never makes a drug homœopathic to any disease; a drug only becomes homœopathic to a case when its symptoms are most similar to the symptoms of that case.

When we are told by the isopathist that these "poisons potentized will *invariably* cure the disease from which they were obtained, EXCEPT when some other miasm is present and obstructs the curative action, notably psora," then we have the entire fabric of isopathic theory swept away. The exception embraces the whole field; for no one ever saw a case of these diseases which was not mixed, and very much mixed, too! To prescribe upon an uncertain diagnosis an unproven remedy in a theoretical manner is not Homœopathy; it is quackery and has not been proven to be successful.

The much vaunted method of Dr. Koch is a shining example of how empirical practice flares up like a burning torch and dies down as quickly, only to leave the world in greater darkness. An allopathic journal recently spoke of his experiments as "a crime" upon mankind. Shall we imitate such experiments?

